# Membrane Trafficking Proteins: A New Target to Identify Resistance to Viruses in Plants

**DOI:** 10.3390/plants10102139

**Published:** 2021-10-09

**Authors:** Aimeric Agaoua, Abdelhafid Bendahmane, Frédéric Moquet, Catherine Dogimont

**Affiliations:** 1 INRAE Génétique et Amélioration des Fruits et Légumes (GAFL), 84140 Montfavet, France; aimeric.agaoua@gmail.com; 2 Institute of Plant Sciences-Paris-Saclay (IPS2), Université Paris-Saclay, INRAE, CNRS, Univ Evry, 91405 Orsay, France; abdelhafid.bendahmane@inrae.fr; 3 Gautier Semences, 13630 Eyragues, France; frederic.moquet@gautiersemences.com

**Keywords:** virus, plant, resistance, vesicle, membrane

## Abstract

Replication cycles from most simple-stranded positive RNA viruses infecting plants involve endomembrane deformations. Recent published data revealed several interactions between viral proteins and plant proteins associated with vesicle formation and movement. These plant proteins belong to the COPI/II, SNARE, clathrin and ESCRT endomembrane trafficking mechanisms. In a few cases, variations of these plant proteins leading to virus resistance have been identified. In this review, we summarize all known interactions between these plant cell mechanisms and viruses and highlight strategies allowing fast identification of variant alleles for membrane-associated proteins.

## 1. Introduction

In agriculture, viruses and their vectors can drastically reduce crop yields by impacting plant development and fruit quality. Nowadays, there is no available treatment to cure infected plants. Thus, insecticides are used to control virus vectors. However, intensive use of insecticides is responsible for severe sanitary and environmental damages [1,2]. With the need for alternative solutions for sustainable agriculture, interest in alternative approaches is increasing [3,4]. Genetic resistance is one of the deployed approaches. Plant resistance is either permitted by the recognition of a viral protein by a plant R-gene-encoded protein, or by a protein modification in plants, preventing viruses from using it for their replication cycle [5]. The development of resistant plants involves the characterization of resistance genes and their introgression into cultivated plants [6,7].

Simple-stranded positive-sense RNA viruses (ssRNA(+)) are the largest group of viruses identified as infecting crops [8,9]. They are obligatory parasites using host cell machinery to assure their replication cycle. Up to now, a small number of genetic resistances targeting essential mechanisms for accumulation of ssRNA(+) viruses have been reported, such as the translation initiation factor eIF4E, the Dynamin-related protein 1 and 2 (DRP1/2) proteins, which are proteins related to endocytosis and cell plate maturation, or PGK2, which is a conserved nucleus-encoded chloroplast phosphoglycerate kinase [10,11,12,13,14].

Recently, published data have revealed the major role played by endomembrane deformation during virus replication in plants [15,16,17]. Viral replication complex (VRC) formation is an essential step for multiplication of ssRNA(+) viruses and takes place in association with the endomembrane [18,19]. As we will see further, the cellular mechanisms of endomembrane trafficking ESCRT, SNARE and COPI/II were shown to be involved in multiplication of ssRNA+ viruses, such as *Potyviruses*, *Tombusviruses* and *Bromoviruses*. This common step of ssRNA+ replication in plant cells could be investigated in order to develop broad-spectrum resistance to viruses. 

In this review, we briefly describe the mechanisms of vesicle formation and associated proteins in plants, especially those from the COPI/II, SNARE, clathrin and ESCRT mechanisms. Then, we discuss known interactions between these plant endomembrane trafficking proteins and viral replication proteins from the genera *Tombusvirus*, *Bromovirus* and *Potyvirus*, which are the most described. Finally, we will discuss the potential use of endomembrane trafficking proteins as targets to identify new alleles conferring virus resistance.

## 2. Plant Proteins Involved in Backward Vesicle Mechanisms

Most of the proteins synthetized in the endoplasmic reticulum (ER) have a function in another localization. The coat protein complex II (COPII) proteins act on the ER to form backward vesicles allowing the exportation of proteins, mainly through the Golgi apparatus (GA), to allow protein maturation [20,21]. Essential compounds required for the formation of vesicles on the ER are the GTPase SAR1, SEC12, the dimer SEC23/SEC24 and the heterodimer SEC13/SEC31 [22,23,24,25,26,27].

The Golgi apparatus (GA) is a major component of protein maturation before they are sent to their functional environment. Proteins in the GA are moved to one cisternae membrane disk to another, thanks to vesicles formed by the coat protein complex I (COPI). COPI is also involved in retrograde transport vesicle formation from the GA to the ER [21,28,29]. Essential components of COPI are ADP-ribosylation factor 1 (ARF1), Golgi brefeldin resistance factor 1 (GBF1), a GTP guanidine-exchange factor, the heteroheptameric coat complex and the cytoplasmic tail of a receptor protein containing a retrieval signal [25,30,31].

The clathrin coat vesicles (CCVs) form the first protein complex involved in vesicle formation that has been observed [32,33]. Nowadays, it remains the most fully understood coat complex in both plants and animals. CCVs can be formed at the trans-face of the GA, plasmatic membrane, vacuole and endosomes in order to target other organelles [34]. Essential components required to induce CCV formation are clathrin heavy chain (CHC) family proteins, chaperone HSC70, the adaptor Fer/Cip4 homology domain-only proteins 1 and 2 (FCHO1/2), the heterotetrameric adaptor protein (AP) complex, the receptor protein and a GTP-binding protein of the ARF family [35]. Large numbers of co-factors are also essential to initiate coat complex recruitment, such as the DRP1 and DRP2 proteins [13,36,37,38].

Since vesicles are free in the cytosol, they have to fuse with the membrane of the targeted organelle. The N-ethylmaleimide-sensitive factor attachment protein receptor (SNARE) proteins are involved in the regulation of the addressing and fusion of vesicles with the targeted organelle [39]. These proteins are localized to the membrane of the vesicle and to the membrane of the destination organelle. Usually, they are anchored to the membrane through a transmembrane domain located in the C-terminal region of the protein. SNARE proteins act in the donor and the receptor partner. v-SNARE proteins on the vesicle of the donor compartment interact with several t-SNARE proteins localized on the receptor organelle. Thus far, 56 members of the SNARE family have been identified in *Arabidopsis thaliana* [24,39,40,41,42,43].

## 3. Plant Proteins Involved in Inward Vesicle Mechanisms

The protein degradation pathway uses backward vesicles to transport proteins from one compartment or plasmatic membrane to the endosome, which is intended to fuse with the lysosome [44]. For their degradation, transmembrane proteins must also move inside the endosome and not remain on the membrane of their compartment. Therefore, an inward vesicle is formed inside the endosome. This structure, called the multivesicular body (MVB), will fuse with the lysosome or vacuole, releasing the vesicle into the lumen of the compartment in order to be degraded [45,46]. The endosomal sorting complex required for transport (ESCRT) is responsible for the formation of inward vesicles during MVB formation. Some proteins of ESCRT have also been involved in cytokinesis [47]. ESCRT is strongly conserved in all eukaryotic organisms and well described in yeasts and mammals [48,49]. It has only recently been described in plants, mainly from homology [50,51]. The ESCRT complex is divided into five small complexes: ESCRT-0, I, II and III and the Vacuolar protein sorting 4 (VPS4) complex. ESCRT-0 is composed of a heterodimer of the proteins VPS27 and HSE1. ESCRT-I is composed of VPS23, VPS28, VPS37 and MVB1. In plants, homologues have been identified, except for MVB1. ESCRT-II is composed of VPS36, VPS22 and two VPS25. ESCRT-III is composed of VPS20, Sucrose non-fermenting 7 (SNF7), VPS24 and VPS2 [52,53,54,55]. Finally, the VPS4 complex is composed of several units of VPS4 and co-factors [56,57,58]. Other co-factors such as BRO1 are essential in ESCRT initiation and recruitment, but their functions are not sufficiently described yet [59].

## 4. Host Intracellular Membrane Association with Viral Replication Complexes

Cellular and molecular events leading to the appearance of symptoms in a plant infected by viruses are still imperfectly known. Viruses take control of host cell mechanisms to their advantage, which probably interferes with plant metabolism and development [60]. Thus, numerous studies have allowed improving the understanding of virus interactions with their hosts [8,61]. The role played by many host factors in each of the stages of viral replication has been described, although many unidentified factors remain. In recent years, the major role of vesicular transport in viral infection has been highlighted [62,63]. Replication of eukaryotic ssRNA(+) is associated with structural changes in intracellular membranes [64]. They form vesicles and quasi-organelles that combine all the elements required for RNA accumulation and for the translation of viral replication proteins, called viral replication complexes (VRCs) [63,65]. VRCs are confined to defined structures, increasing the replication efficiency and preventing the activation of cellular defense mechanisms such as double-stranded RNA recognition during replication [18,62]. Membranes are an integral part of VRC formation. Their origins and their final destinations are different depending on the virus. For instance, carnation Italian ring spot virus (CIRV; genus *Tombusvirus*; family *Tombusviridae*) induces vesicles derived from the external membrane of mitochondria, and tobacco mosaic virus (TMV; genus *Tobamovirus*; family *Virgaviridae*) induces vesicles derived from the tonoplasts [66,67,68]. Studies seem to show that most cellular compartments can be a target for VRC formation but that each virus uses a defined host compartment [69].

The essential character of ssRNA(+) VRC anchoring in the membrane has been repeatedly shown in yeast by knock-out approaches of endomembrane trafficking mechanisms [70,71,72,73,74]. In plants, transient inhibition approaches of one gene of each of these mechanisms have shown the same reduction in the efficiency of viral accumulation [75,76]. Thanks to the development of membrane-based interaction assays in plant–virus protein interactions, several interactions between membrane-associated viral proteins and membrane trafficking proteins have been revealed in the last decade in plants. In Table 1, we regroup most of the data of interactions between a host membrane trafficking protein with viral proteins and approaches used to demonstrate the impact on viral accumulation in the host. In the following sections, we will detail these data and what we know about viral vesicles for the three major ssRNA(+) plant virus genera.

## 5. Membrane Deformation and Involved Proteins during Replication of *Tombusviruses*

Tomato bushy stunt virus (TBSV), cucumber necrosis virus (CNV) and cymbidium ringspot virus (CymRSV; genus *Tombusvirus*; family *Tombusviridae*) induce membrane deformations at the peroxisome level in plants and yeasts [88,89,90,91,92]. They form spherule-like vesicles, similar to inward vesicles of MVBs, but maintaining a neck opening to the cytosol [93]. The modified peroxisomes contain host and viral replication proteins and viral RNA. These vesicles appear to relocate from peroxisomes to de novo peroxisomes derived from the endoplasmic reticulum (pERs) [91]. Mechanisms associated with TBSV movement from peroxisomes to pERs are not know. It has been observed that VRCs of TBSV are delocalized to the endoplasmic reticulum without affecting the efficiency of viral accumulation, when the peroxisome is absent in yeasts that do not express Peroxisomal biogenesis factor 3 or 19 (PEX3/19) [94]. Differently, the *Tombusvirus* carnation Italian ring spot virus (CIRV) induces the formation of MVB-like vesicles in the mitochondrial membrane [66]. The bipartite red clover necrotic mosaic virus (RCNMV; genus *Dianthovirus*; family *Tombusviridae*) induces membrane modifications and viral protein accumulation in the endoplasmic reticulum [95]. The kinetics of events during the formation of *Tombusvirus* VRCs are still unknown. However, the role of ESCRT in virus-induced distortion has been repeatedly shown. The SNARE mechanism also appears to be involved in the targeting of viral vesicles from peroxisomes to pERs [16,76,77,84].

The p33 protein of *Tombusvirus* is a transmembrane protein essential for the formation of VRCs. It is involved in a large number of interactions with other viral replication proteins and host proteins [92,96]. The interaction of the p33 protein with the ESCRT proteins VPS23, VPS24, VPS20, VPS2 and VPS appears to be responsible for the formation of inward vesicles at peroxisomes, pERs and the ER [75,76,77,78]. An interaction of p33 with VPS34 and the Bro1 accessory protein has also been shown to be involved in the regulation of the ESCRT complex [77,79]. The p33 protein carries a targeting signal peptide responsible for the induction of COPI vesicle formation through interaction with the ARF1 protein. Inhibition of the protein interaction COPI–ARF1 inhibits the movement of viral vesicles from peroxisomes to pERs [90]. A direct interaction of TBSV p33 with SNARE UFE1 and USE1 has been shown [16]. Their deletion delocalizes VRCs to the ER membrane and reduces TBSV replication in yeasts and plants [16]. During CIRV replication, the protein responsible for the formation of VRCs has an additional domain. This protein, called p36, is responsible for VRC localization in mitochondria, in contrast with *Tombusviruses* encoding a p33 [66,97]. Up to now, only the interaction of VPS23 with the p36 protein of CIRV has been shown [76]. Loss of activity of most of these susceptibility factors by deletion of membrane trafficking proteins in yeasts or over-expression of dominant-negative mutants in plant leaves led to a significant reduction in RNA accumulation [71,75,76,77,78].

## 6. Membrane Deformation and Involved Proteins during Replication of *Potyviruses*

*Potyviridae* is the largest family of plant-infecting viruses [98]. This virus family cannot infect yeasts. Therefore, studies on proteins involved in the replication of these viruses mostly rely on transient inhibition of essential characters of plant membrane trafficking mechanisms and punctual mutation. Turnip mosaic virus (TuMV; genus *Potyvirus*; family *Potyviridae*) is the main model used to describe the vesicle formation in infected cells by *Potyviruses*. Viral replication of several *Potyviruses* has been localized to chloroplasts. *Potyvirus* membrane deformation in chloroplasts is characterized by the formation of a large compartment called the cytoplasmic inclusion (CI) body [99]. VRC assembly occurs in the ER compartment, and then VRCs are moved inside vesicles from the ER to chloroplasts [100,101,102]. The function of the CI body in chloroplasts is not clear. In contrast to *Tombusviruses*, the replication of TuMV occurs necessarily in chloroplasts. Vesicle translocation from the ER to chloroplasts is required for *Potyviruses* to succeed in replication [83,103]. For ssRNA(+) viruses which form endoplasmic reticulum VRCs and are addressed to another site, an interesting model was shown by H. Sanfaçon and J. Laliberté [69]. In this model, the authors suggest that a first budding event within the ER lumen is followed by a second budding event, allowing the formation of a second membrane, and upon detachment from the ER, this gives rise to a double-membrane vesicle in the cytoplasm [104]. Viral vesicles holding TuMV VRCs were also identified in the MVB compartment, which are released into the apoplast by fusion of the MVB to the plasmatic membrane [105,106]. 

The 6K2 protein of *Potyviruses* is a transmembrane protein responsible for ER-induced vesicles and for chloroplast targeting [107]. The role of a second transmembrane protein encoded by the *Potyvirus* genome, called 6K1, is still poorly understood [108,109]. The vesicle formation occurring in ER requires, at least, the recruitment by direct interaction of the COPII protein SEC24a by 6K2 [83,110]. COPII vesicles are backward vesicles; thus, following the double-membrane vesicle model, another membrane trafficking mechanism should be involved in the first inward vesicle. For now, the only information supporting this model is a mutation in the ESCRT protein VPS4, involved in inward vesicles, which has been shown to induce resistance to the zucchini yellow mosaic virus (ZYMV, genus *Potyvirus*) in cucumber [85]. Then, the COPII vesicle, formed at the Golgi apparatus, is transported to the chloroplasts thanks to the interaction of 6K2 with Vesicle transport v-SNARE 11 (VTI11), an essential protein for TuMV replication [81]. A second SNARE protein, Syntaxin-71 (SYP71), is also involved in viral vesicle transport due to an indirect interaction between 6K2 and the SNARE co-factor Vesicle-associated protein 27 (VAP27) [81,82,83]. This interaction seems to be involved in MVB addressing of the viral vesicle [106]. Involvement of clathrin coat vesicles (CCV) in ssRNA(+) virus replication has not been shown, except for TuMV. Recently, the interaction of the CCV DRP1 and DRP2 proteins with the 6K2, Viral protein genome-linked (VPg), Capsid protein (CP) and Cylindrical inclusion (CI) viral replication proteins was shown to be essential to TuMV replication [14,111]. However, the mechanisms associated with DRP1/2 recruitments are not known. 

## 7. Membrane Deformation and Involved Proteins during Replication of *Bromoviruses*

In plant and yeast cells, replicating Brome mosaic virus (BMV; genus *Bromovirus*; family *Bromoviridae*) RNAs occurs at the outer perinuclear endoplasmic reticulum (ER) membrane which is invaginated towards the lumen [112,113]. The BMV 1a protein serves as the primary organizer to form active replication compartments: it invaginates the outer ER membranes into the ER lumen to form spherules, recruits RNA templates into the interior of preformed spherules by recognizing the cis-element RE present only in viral genomic RNAs and also interacts with and recruits specific host factors to the site of viral replication [114,115,116].

Deletion of seven ESCRT proteins, VPS12, VPS20, SNF7, VPS24, VPS2, VPS4 and DID2 (VPS46), in yeasts induces a significant reduction in BMV RNA replication. Moreover, deletion of SNF7 leads to a total inhibition of RNA replication. Further investigation revealed a direct interaction of SNF7 with 1a [72]. BMV replication is also linked to the host reticulon homology domain protein (RHP) family. These proteins have been characterized in compartment shapes, which differ from a spherical shape-like ER [117,118]. Deletion of the three more expressed RHP protein reticulons 1 and 2 (RTN1/2) and YOP1 reduces BMV accumulation [86]. The mechanisms involved are not well understood. A genetic resistance to cucumber mosaic virus (CMV, genus *Cucumovirus*; family *Bromoviridae*) was shown to be based on the SNARE protein VPS41. A single polymorphism led to a significant reduction in CMV accumulation in melon [119]. For now, the role of the SNARE complex in BMV replication is not known.

## 8. Challenges and Opportunities for the Future

**Figure 1:**
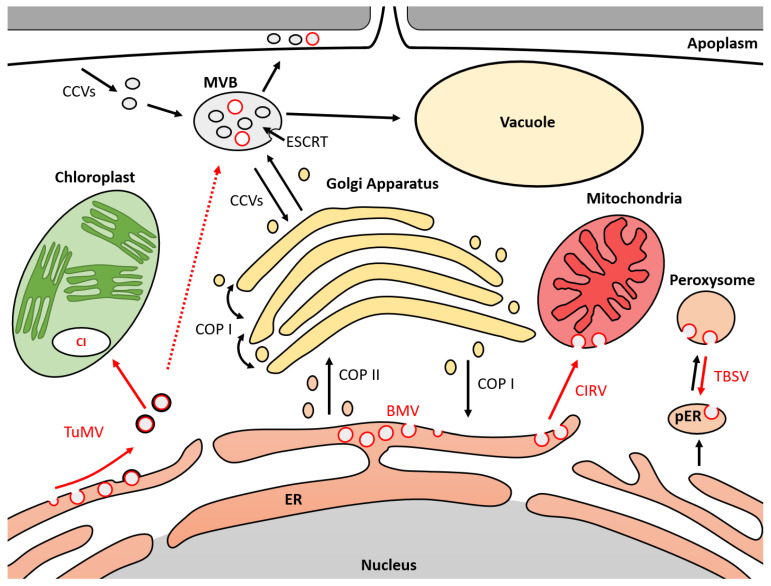
Model of membrane trafficking mechanisms hijacked by ssRNA(+) viruses during infection. MVB (multivesicular body); ER (endoplasmic reticulum); pER (derived endoplasmic reticulum for peroxisome biogenesis); ESCRT (endosomal sorting complex required for transport); COPI/II (coated protein I/II); CI (cytosolic inclusion); CCVs (clathrin-coated vesicles); TBSV (tomato bushy stunt virus); CIRV (carnation Italian ring spot virus); TuMV (turnip mosaic virus); BMV (Brome mosaic virus). Black circles represent cell vesicles. Red circles represent viral vesicles. Black arrows represent movement of cell vesicles. Red arrows represent movement of viral vesicles. Dotted red arrows represent hypothetical vesicle movement.

In this review, we showed that several endomembrane trafficking proteins are hijacked by ssRNA(+) viruses during plant infection (Table 1). Resulting viral vesicles have been localized in different cellular compartments (Figure 1). Thus, several SNARE and ESCRT proteins are involved in the replication of tomato bushy stunt virus (TBSV). Viral vesicles were observed in the lumen of peroxisomes, which are connected to the cytoplasm via a neck-like structure. These vesicles also appear to move towards pre-peroxisomes derived from the ER. During the replication of Brome mosaic virus (BMV), ESCRT proteins were shown to be involved in the formation of vesicles and were observed at the level of the endoplasmic reticulum (ER). This testifies to the diversion of the initial activity of the ESCRT from the multivesicular bodies (MVBs) to the ER. The direct interaction between the ESCRT-III SNF7 protein and BMV has been shown to be essential for virus replication. The role of ESCRT-I and ESCRT-II in viral vesicle formation suggests the possible involvement of additional ESCRT proteins or co-factors. In *Potyviruses*, turnip mosaic virus (TuMV) is the main model used to describe the formation of vesicles in the cells of infected plants. Viral vesicles have been localized in the ER, chloroplasts, MVBs, vacuoles and apoplasm [69]. The 6K2 protein of TuMV has been shown to be responsible for the externalization of viral vesicles localized in the ER by an interaction with the COPII SEC24a protein. These vesicles are derived from the COPII pathway. SNARE proteins VTI11 and SEC22 appear to be involved in addressing viral vesicles to MVBs and vacuoles. However, the mechanisms associated with the internalization of vesicles in the ER and MVBs are not known. The ESCRT is the only described mechanism associated with the formation of luminal vesicles. Cell localizations for these vesicles are represented in Figure 1.

The identification by protein interaction approaches of a large number of susceptibility factors required for vesicle formation during virus infection in plants opens up new opportunities to identify new resistance genes. Thus, deletion or modification of the amino acids of the proteins encoded by nine of these genes could be used for resistance to *Tombusvirus*; five genes are candidates for resistance to *Potyvirus* and two for resistance to *Bromovirus* (Table 1). Changes in two of these susceptibility factors, VPS41 and VPS4, have already been identified as responsible for reduced virus accumulation in plants [85,87]. Taken together, these data point towards a common mechanism of infection for ssRNA+ viruses, suggesting that loss of function of these genes could confer broad-spectrum resistance in ssRNA+ viruses. Moreover, the translational potential of these genes could accelerate studies on model organisms and the identification of new resistance mechanisms in crop species [21,34,120].

Interestingly, viral proteins involved in the recruitment of membrane trafficking proteins, p33 for *Tombusvirus*, 6K2 for *Potyvirus* or 2a for *Bromovirus*, are membrane-associated proteins [89,121,122]. Some interaction assays are well suited to the identification of membrane-bound protein complexes. For example, split-ubiquitin yeast two-hybrids (Su-Y2H) or bimolecular fluorescent complementation (BiFC or Split-YFP) offer the opportunity of reliably identifying new genes of interest [123]. Conservation of these mechanisms across organisms, such as yeasts, also offers a rapid approach to identifying new interactions using viruses that infect both plants and yeasts, as it has been widely described for TBSV and BMV. In addition, efficient genetic tools are available such as TILLING (Targeting Induced Local Lesions in Genomes) and Eco-TILLING (from natural diversity), which allow new alleles to be identified [124].

In conclusion, these different steps should open the door, in a short time, to the pyramiding of new resistance genes and to the construction of genetic resistances based on new mechanisms, such as those involved in membrane trafficking proteins during viral infection.

## Figures and Tables

**Table 1:** Endomembrane trafficking proteins involved in virus replication. TBSV (tomato bushy stunt virus); CIRV (carnation Italian ring spot virus); RCNMV (red clover necrotic mosaic virus); TuMV (turnip mosaic virus); ZYMV (zucchini yellow mosaic virus); BMV (brome mosaic virus); CMV (cucumber mosaic virus).

Virus	Viral Protein	Host	Host Protein	Mechanisms	Relation between Pathogen and Host	Ref.
*Tombusvirus*						
TBSV	p33	*A. thaliana*	VPS23, BRO1	ESCRT	Protein interaction	[75]
TBSV	p33	Yeast	VPS4, VPS24	ESCRT	Protein interaction	[77]
TBSV	p33	Yeast	VPS23	ESCRT	Protein interaction	[78]
TBSV	p33	Yeast	UFE1, USE1	SNARE	Protein interaction	[16]
TBSV	p33	Yeast	VPS34	ESCRT	Protein interaction	[79]
TBSV	-	Yeast	VPS15, VPS30, VPS34	ESCRT	KO reducing viral replication	[79]
TBSV	-	Yeast	VPS18, VPS32, VPS24, VPS29, VPS4, VPS41, DID2, VPS23, VPS28, VPS51, VPS61, VPS69	ESCRT, SNARE	KO reducing viral replication	[77]
TBSV, CIRV	-	*A. thaliana*	VPS4	ESCRT	Dominant-negative reducing viral replication	[76]
CIRV	p36	Yeast	VPS23	ESCRT	Protein interaction	[76]
TBSV	p33	Yeast	PEX19		Protein interaction	[74]
RCNMV	p27	In vitro	ARF1	COPI	Protein interaction	[80]
*Potyvirus*					Protein interaction	
TuMV	6K2	In vitro	VTI11	SNARE	Protein interaction	[81]
TuMV	6K2	Yeast	VAP27	SNARE	Protein interaction	[82]
TuMV	6K2	Yeast	SEC24a	COPII	Protein interaction	[83]
TuMV	6K2, VPg, CP, CI	*A. thaliana*	DRP1/2	CCV	Protein interaction	[77,84]
ZYMV	-	*Cucumis sativus*	VPS4	ESCRT	Substitution inducing resistance	[85]
*Bromovirus* and *Cucumovirus*						
BMV	1a	In vitro	SNF7	ESCRT	Protein interaction	[72]
BMV	1a	In vitro	RTN1p, RTN2p, YOP1p	RHP	KO reducing viral replication	[86]
BMV	1a	Yeast	VPS23, VPS20, SNF7, VPS24, VPS2, VPS4, DID2, VPS60	ESCRT	KO reducing viral replication	[72]
CMV		*Cucumis melo*	VPS41	SNARE	Substitution inducing resistance	[87]

## Data Availability

All relevant data are within the paper.
